# Atypical histological abnormalities in an adult patient with nephronophthisis harboring *NPHP1* deletion: a case report

**DOI:** 10.1186/s12882-021-02466-z

**Published:** 2021-07-10

**Authors:** Maiko Akira, Hitoshi Suzuki, Arisa Ikeda, Masako Iwasaki, Daisuke Honda, Hisatsugu Takahara, Hisaki Rinno, Shigeki Tomita, Yusuke Suzuki

**Affiliations:** 1 Department of Nephrology, Juntendo University Urayasu Hospital, 2-1-1 Tomioka, Urayasu-shi, Chiba, 279-0021 Japan; 2 Department of Pathology, Juntendo University Urayasu Hospital, Chiba, Japan; 3 Department of Nephrology, Juntendo University Faculty of Medicine, Tokyo, Japan

**Keywords:** Nephronophthisis, Distal tubule, Renal biopsy, End-stage renal failure

## Abstract

**Background:**

Nephronophthisis (NPHP) is a chronic tubular interstitial disorder that exhibits an autosomal recessive genetic form and causes progressive renal failure in children. Patients with NPHP rarely show urinary abnormalities, edema, or hypertension. Thus, NPHP is often detected only when renal failure becomes advanced. NPHP can be divided into three types based on the age of end-stage renal failure, i.e., infant type (approximately 5 years old), juvenile type (approximately 13–14 years old), and adolescent type (approximately 19 years old). Here, we report a case of NPHP diagnosed by genetic analysis at 26 years of age with atypical histological abnormalities.

**Case presentation:**

A 26-year-old woman showed no growth disorders or urinary abnormalities in annual school physical examinations. However, at a check-up at 26 years old, she exhibited renal dysfunction (eGFR 26 mL/min/1.73 m^2^). Urine tests indicated low specific gravity of urine, but not proteinuria or microscopic hematuria. Urinary β2-microglobulin was high (805 μg/L), and renal biopsy was performed for definitive diagnosis. Histological findings showed no significant findings in glomeruli. However, moderate fibrosis was observed in the interstitial area, and moderate atrophy was observed in the tubules. There were no significant findings in immunofluorescence analysis, and no electron dense deposits were detected by electron microscopy. Although cyst-like expansion of the tubules was unclear, tubular atrophy was dominantly found in the distal tubule by cytokeratin 7 staining. Genetic analysis of the *NPHP1* gene showed complete deletion of this gene, leading to a definitive diagnosis of NPHP.

**Conclusions:**

NPHP is not merely a pediatric disease and is relatively high incidence in patients with adult onset end-stage of renal disease. In this case, typical histological abnormalities, such as cyst-like expansion of the tubular lesion, were not observed, and diagnosis was achieved by genetic analysis of the *NPHP1* gene, which is responsible for the onset of NPHP. In patients with renal failure with tubular interstitial disease dominantly in the distal tubules, it is necessary to discriminate NPHP, even in adult cases.

## Background

Nephronophthisis (NPHP) is a chronic tubular interstitial injuries and is the most common genetic causes of renal failure in children and young adults [1]. The estimated incidence of NPHP is 1:50,000 to 1:70,000 [2, 3], and account for approximately 10 to 15% of end-stage of renal disease (ESRD). Clinical features of NPHP are usually not specific, thus common features of chronic kidney disease (CKD), such as, urinary abnormalities, edema, and hypertension are rarely observed in patients with NPHP. Impairment ability to concentrate urine and retain body fluid induce polyuria, polydipsia, and decreased maximum urine concentration. Several patients with NPHP show extrarenal abnormalities, such as cerebellar ataxia, liver fibrosis, situs inversus, abnormal skeleton and facial features, Senior-Loken syndrome, Cogan syndrome and Joubert syndrome [4, 5].

NPHP is divided into three types based on the onset of ESRD, viz., infant type (approximately 5 years old), juvenile type (approximately 13–14 years old), and adolescent type (approximately 19 years old) [6]. It is not easy to diagnose as NPHP because of lack of specific clinical symptom. Thus, many of patients with NPHP progresses to ESRD in childhood or early adolescence. The diagnosis of NPHP is usually made by genetic analysis, as the findings in renal biopsy are not specific. The histopathology of NPHP is characterized by tubulointerstitial abnormalities, such as tubular atrophy, thickening or thinning of the tubular membrane, interstitial fibrosis and inflammation. However, those pathological phenotypes are not disease specific. Therefore, it is important to perform genetic analysis in suspected NPHP patients. Although NPHP has genetic heterogeneity, the most common NPHP mutation is a homozygous deletion of *NPHP1*, which is identified in 20% of patients with NPHP harboring *NPHP* gene mutations [7, 8]. Overall, a greater consideration of the diagnosis of NPHP is necessary to prevent expedited decline of renal function.

In the current report, we describe a case of NPHP diagnosed by genetic analysis at 26 years of age in a patient with atypical histological abnormalities.

## Case presentation

The patient was a 26-year-old Japanese woman who showed no growth disorders or urinary abnormalities at school check-ups. However, during a medical check-up at 26 years of age, she showed renal dysfunction (serum creatinine 2.2 mg/dL). The estimated glomerular filtration rate (eGFR) was 26 mL/min/1.73 m^2^, and urine tests indicated low specific gravity of urine (1.004); however, proteinuria and microscopic hematuria were not detected. Urinary β2-microglobulin (β2-MG) was high (805 μg/L; Table 1). Analysis of her family history revealed that her father had chronic myelogenous leukemia and her mother had Sjogren’s syndrome and renal dysfunction. At the time of renal biopsy, the patient’s height, weight, and blood pressure were 155.6 cm, 51.4 kg, and 122/91 mmHg, respectively. Physical examination findings were normal. An abdominal computed tomography scan revealed that both kidneys were normal in size. We performed a percutaneous renal biopsy for definitive diagnosis.

**Table 1:** Clinical examination at the renal biopsy

Hematological		Reference values		Blood biochemistry		Reference values		Immunological test		Reference values	
WBC	5700	3300 ~ 8600	/μl	TP	7.0	6.6 ~ 8.1	g/dl	CH50	59	25.0 ~ 48.0	U/μl
Hb	10.2	11.6 ~ 14.8	g/dl	Alb	4.2	4.1 ~ 5.1	g/dl	C3	99	86 ~ 160	mg/dl
Plt	28.2	15.8 ~ 34.8	× 10^4^/μl	BUN	19	8.0 ~ 20.0	mg/dl	C4	24	17 ~ 45	mg/dl
				Cre	2.03	0.46 ~ 0.79	mg/dl	IgG	1216	870 ~ 1700	mg/dl
**Urinalysis**				eGFR	26	60~	mL/min/1.73m^2^	IgA	228	110 ~ 410	mg/dl
pH	7.335	5.0 ~ 8.0		Cystatin C	2.05	0.56 ~ 0.87	mg/dl	IgM	152	33 ~ 190	mg/dl
Specific gravity	1.004			Na	141	138 ~ 145	mM/l	ANA	<× 40	~ 40	
Protein	< 20		mg/dl	K	4.1	3.6 ~ 4.8	mM/l	anti SSA-Ab	1.9	~ 7	U/ml
RBC	< 1		/HPF	Cl	107	101 ~ 108	mM/l	anti SSB-Ab	< 1.0	~ 7	U/ml
WBC	< 1		/HPF	Ca	8.5	8.8 ~ 10.1	mg/dl	MPO-ANCA	< 1.0	~ 3.5	U/ml
U-NAG	1.4	0.7 ~ 11.2	μg/l	CRP	< 0.1	0.00 ~ 0.14	mg/dl	PR3-ANCA	< 1.0	~ 3.5	U/ml
U-β2MG	805	~ 230	μg/l	HbA1c	5.7	4.9 ~ 6.0	%	anti GMB-Ab	< 2.0	~ 3	U/ml

**Fig. 1:**
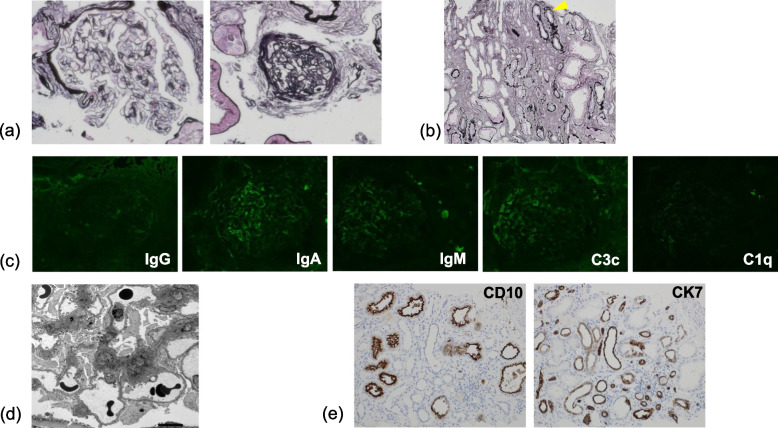
Renal biopsy histopathology. **a** Light microscopic findings indicate global sclerosis in half of the glomeruli. However, the other glomeruli showed minor lesions (periodic acid-silver methenamine stain; original magnification, 200×). **b** Moderate fibrosis was observed in the interstitial area, and moderate atrophy was observed in the tubules (periodic acid-silver methenamine stain; original magnification, 100×). Irregular splitting TBM was also observed in some tubules (arrowhead). **c** There were no significant findings in immunofluorescence analysis. **d** No electron dense deposits were detected by electron microscopy. **e** Cyst-like expansion of the tubular was unclear. Tubular atrophy was dominantly found in the distal tubule by CK7 staining (original magnification, 100×)

Histological findings showed global sclerotic lesions in approximately half of glomeruli (Fig. 1a). Moderate fibrosis was observed in the interstitial area, and moderate atrophy was observed in the tubules. Irregular splitting of the tubular basement membrane (ISTBM) [9] was also observed in some tubules (Fig. 1b). There were no significant findings in immunofluorescence analysis (Fig. 1c), and no electron dense deposits were detected by electron microscopy (Fig. 1d). Although cyst-like expansion of the tubules was unclear, tubular atrophy was dominantly found in the distal tubules by cytokeratin 7 (CK7) staining (Fig. 1e).

**Fig. 2:**
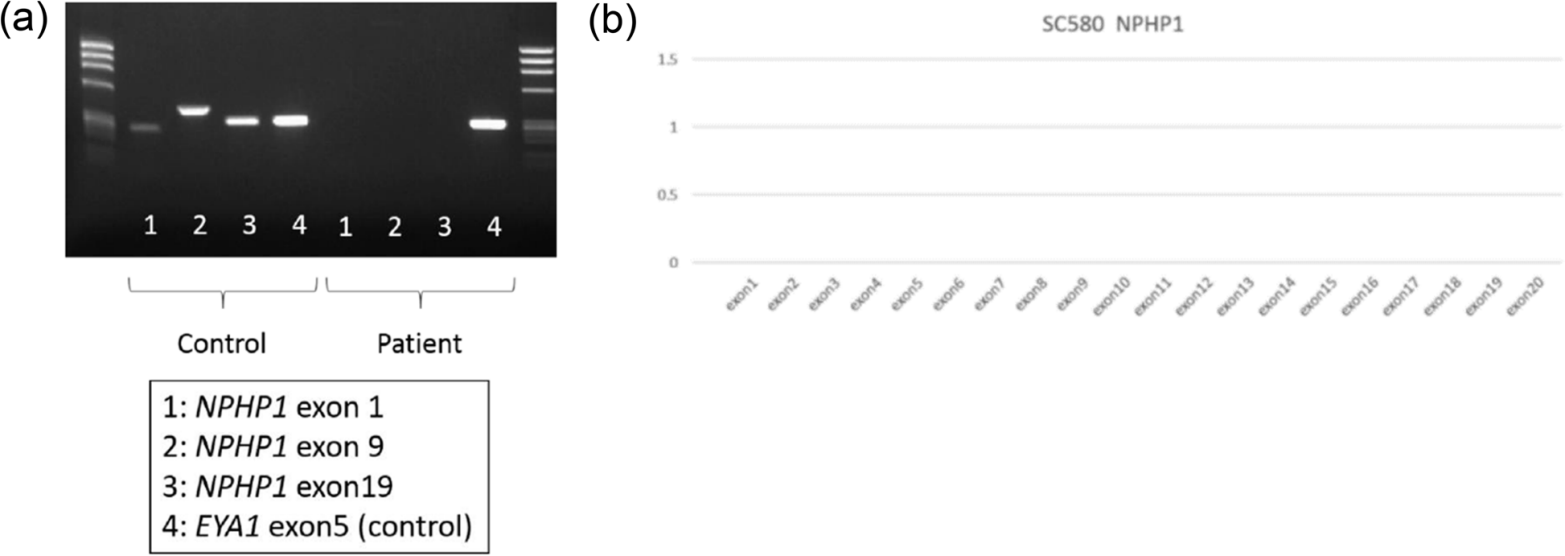
Genetic analysis of the *NPHP1* gene. **a** No exons from *NPHP1* were amplified. **b** Multiplex ligation-dependent probe amplification analysis indicated complete deficiency of the *NPHP1* gene

Next, we analyzed the *NPHP1* gene, the most common gene responsible for NPHP. No exons of *NPHP1* (1, 9, and 19) were amplified (Fig. 2a). Furthermore, multiplex ligation-dependent probe amplification analysis indicated complete deficiency of the *NPHP1* gene (Fig. 2b). Based on these results, both alleles of *NPHP1* were considered to be deleted in the current case, leading to a diagnosis of a total deletion of *NPHP1*.

## Discussion and conclusion

NPHP is the most common genetic cause of kidney failure in children and young adults [8, 10], with an estimated incidence of 1:50,000 [11, 12]. NPHP accounts for 6–10% of cases of kidney failure and 15% of renal transplants among children [13, 14]. NPHP has significant genetic heterogeneity, with biallelic pathogenic variants in 20 different genes identified as causative [15] with *NPHP1* (20%) being the most common gene [8, 16, 17]. Although NPHP is the most common genetic cause of chronic kidney disease (CKD) in children, its prevalence in the adult population is thought to be low. However, Snoek R, et al. recently reported that the frequency of NPHP in adult onset ESRD is substantially higher than previous report [18]. Genome-wide association study using adult renal transplant recipients from several cohorts revealed median age of adult onset of ESRD was 30, ranged from 18 to 61 years old, in patients with NPHP [18]. Thus, NPHP should be considered as a differential diagnosis in adult patients with unidentified ESRD. With no specific management available, treatment focuses on supportive and preventative strategies to preserve kidney function. However, a greater consideration of the diagnosis of NPHP in adult patients with kidney failure and progressive CKD is required to allow informed prognosis, targeted screening of at-risk family members, and expedited preventative and management strategies of kidney function decline [19, 20].

The findings of this case report emphasize the importance of CD10 and CK7 staining in cases in which the cause of renal dysfunction is unclear. Irregular splitting TBM in tubules is an important finding suggesting autosomal dominant tubulointerstitial kidney disease [9]. In patients with renal failure with tubular interstitial disease dominantly in the distal tubule, it is necessary to discriminate NPHP, even in adult patients. Moreover, it is important to perform genetic testing to obtain a definitive diagnosis, which will enable appropriate genetic counseling and treatment. Further studies are necessary to clarify the mechanisms leading to wide variance in age of disease onset.

In summary, NPHP often progresses to ESRD at an average age of 13–14 years old. However, NPHP is not merely a pediatric disease and is relatively high incidence in patients with adult onset ESRD. Even in cases without typical histological abnormalities, such as cyst-like expansion of tubular lesions, differential diagnosis of NPHP is critical. Thus, wider application of genetic testing is recommended to patients with unidentified ESRD.

## Data Availability

The datasets used in the current study are available from the corresponding author on reasonable request.

## Abbreviations

NPHP: Nephronophthisis
ESRD: End-stage of renal disease
eGFR: Estimated glomerular filtration rate
β2-MG: β2-microglobulin
TBM: Tubular basement membrane
CK7: Cytokeratin 7
