# Correction: Atypical histological abnormalities in an adult patient with nephronophthisis harboring NPHP1 deletion: a case report

**DOI:** 10.1186/s12882-023-03352-6

**Published:** 2023-11-07

**Authors:** Maiko Akira, Hitoshi Suzuki, Arisa Ikeda, Masako Iwasaki, Daisuke Honda, Hisatsugu Takahara, Hisaki Rinno, Shigeki Tomita, Yusuke Suzuki

**Affiliations:** 1 Department of Nephrology, Juntendo University Urayasu Hospital, 2-1-1 Tomioka, Urayasu-Shi, Chiba 279-0021 Japan; 2 Department of Pathology, Juntendo University Urayasu Hospital, Chiba, Japan; 3 Department of Nephrology, Juntendo University Faculty of Medicine, Tokyo, Japan

**Correction: Nephrology 22, 261 (2021)**

**https://doi.org/10.1186/s12882-021-02466-z**

Following publication of the original article [1], we found that there were misprint in the Fig. 2a. The correct Fig. 2 is given below:

**Fig. 2:**
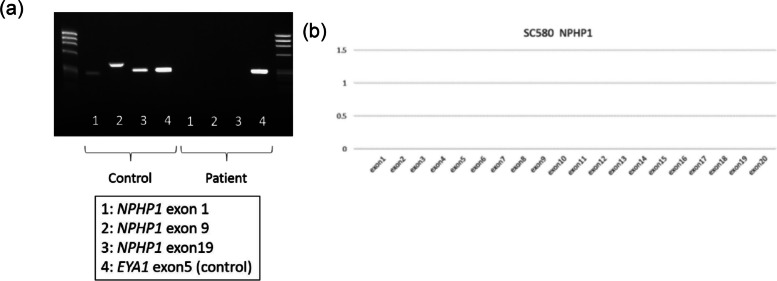
Genetic analysis of the NPHP1 gene. **a** No exons from NPHP1 were amplified in this patient. **b** Multiplex ligation-dependent probe amplification analysis indicated complete deficiency of the NPHP1 gene

The original article has been corrected.
